# Preclinical optimization of Ly6E-targeted ADCs for increased durability and efficacy of anti-tumor response

**DOI:** 10.1080/19420862.2020.1862452

**Published:** 2020-12-31

**Authors:** Josefa Dela Cruz Chuh, MaryAnn Go, Yvonne Chen, Jun Guo, Hanine Rafidi, Danielle Mandikian, Yonglian Sun, Zhonghua Lin, Kellen Schneider, Pamela Zhang, Rajesh Vij, Danielle Sharpnack, Pamela Chan, Cecile de la Cruz, Jack Sadowsky, Dhaya Seshasayee, James T. Koerber, Thomas H. Pillow, Gail D. Phillips, Rebecca K Rowntree, C. Andrew Boswell, Katherine R. Kozak, Andrew G. Polson, Paul Polakis, Shang-Fan Yu, Peter S. Dragovich, Nicholas J. Agard

**Affiliations:** a Departments of Biochemical and Cellular Pharmacology, Genentech Inc, South San Francisco, CA, USA; b Research biology, Genentech Inc, South San Francisco, CA, USA; c Antibody Engineering, Genentech Inc, South San Francisco, CA, USA; d Preclinical & Translational Pharmacokinetics and Pharmacodynamics, Genentech Inc, South San Francisco, CA, USA; e Protein Chemistry, Genentech Inc, South San Francisco, CA, USA; f Discovery Chemistry, Genentech Inc, South San Francisco, CA, USA

**Keywords:** Antibody-drug conjugate, Ly6E, virus-like particles, ADC resistance, antibody discovery

## Abstract

Early success with brentuximab vedotin in treating classical Hodgkin lymphoma spurred an influx of at least 20 monomethyl auristatin E (MMAE) antibody-drug conjugates (ADCs) into clinical trials. While three MMAE-ADCs have been approved, most of these conjugates are no longer being investigated in clinical trials. Some auristatin conjugates show limited or no efficacy at tolerated doses, but even for drugs driving initial remissions, tumor regrowth and metastasis often rapidly occur. Here we describe the development of second-generation therapeutic ADCs targeting Lymphocyte antigen 6E (Ly6E) where the tubulin polymerization inhibitor MMAE (Compound **1**) is replaced with DNA-damaging agents intended to drive increased durability of response. Comparison of a *seco*-cyclopropyl benzoindol-4-one (CBI)-dimer (compound **2**) to MMAE showed increased potency, activity across more cell lines, and resistance to efflux by *P*-glycoprotein, a drug transporter commonly upregulated in tumors. Both anti-Ly6E-CBI and -MMAE conjugates drove single-dose efficacy in xenograft and patient-derived xenograft models, but *seco-*CBI-dimer conjugates showed reduced tumor outgrowth following multiple weeks of treatment, suggesting that they are less susceptible to developing resistance. In parallel, we explored approaches to optimize the targeting antibody. In contrast to immunization with recombinant Ly6E or Ly6E DNA, immunization with virus-like particles generated a high-affinity anti-Ly6E antibody. Conjugates to this antibody improve efficacy versus a previous clinical candidate both *in vitro* and *in vivo* with multiple cytotoxics. Conjugation of compound **2** to the second-generation antibody results in a substantially improved ADC with promising preclinical efficacy.

## Introduction

Lymphocyte antigen 6E (Ly6E) is an interferon-inducible glycophosphatidylinositol-linked glycoprotein expressed on the surface of multiple solid tumors.^1^ In combination with its receptor syncytin-A, Ly6E is thought to promote membrane fusion and plays an essential role in connecting the placenta to the developing fetus.^2^ In adulthood, low-level expression of Ly6E persists in some tissues, providing an essential receptor for infection by multiple viruses.^3^ In contrast to this baseline expression, Ly6E is highly expressed in a subset of breast, lung, colon, ovarian, pancreatic, kidney and gastric carcinomas.^1,4^ The immediate impact of this overexpression is not well established, but it may drive cell growth and angiogenesis through the hypoxia-inducible factor 1 pathway.^5^ Due to its differential expression between cancer and normal tissue, Ly6E is considered a promising target for antibody–drug conjugate (ADC) development. Previously, an anti-Ly6E-monomethyl auristatin E (MMAE) conjugate (DLYE5953A) demonstrated proof of therapeutic concept in preclinical models,^1^ and results from a Phase 1 clinical study indicate eight of 68 patients had confirmed partial responses.^6,7^ While encouraged by these initial results, post-progression biopsies on three patients all showed upregulation of *P*-glycoprotein (Pgp) while high levels of Ly6E expression were maintained. Given evidence of therapeutic efficacy, combined with lack of durability potentially driven by drug exporters, we sought to further optimize the Ly6E-targeted ADCs via conjugation of alternative (non-tubulin binding) payloads.

ADCs can be enhanced in multiple ways, including by improving the antibody to enable enhanced binding, internalization, or epitope-specific effects (e.g., blocking antibodies, antibody-dependent cytotoxicity), or by improving the linker-drug to enable better tolerability, more effective tumor killing, and/or reduced susceptibility to resistance mechanisms (e.g., upregulation of efflux pumps or compensatory biological pathways).^8^ Because the discovery campaigns leading to the antibody component of the clinical ADC identified only a single clone with detectable binding by flow cytometry (anti-Ly6E, also called 9B12), we anticipated that additional antibodies might prove to be more efficacious.^1^ The initial campaigns, however, were limited, primarily due to challenges of generating high-quality recombinant Ly6E.

While immunization with recombinant protein remains the most common approach to generate therapeutic antibodies, alternative approaches have been explored, including DNA immunizations using electroporation or gene guns,^9^ whole-cell immunizations, and immunizations with virus-like particles (VLPs).^10^ In each of the alternatives, the antigen is presented to the immune system in its native (membrane-associated) context facilitating development of antibodies targeting the well-folded target. The facility of generating well-behaved antigen must be weighed against the often-weak immunogenicity of DNA-based immunizations and immune responses to undesired cellular- or vesicle-associated antigens. In particular, cell- or VLP-based immunizations require deeper levels of characterization to ensure target specificity.

Beyond improvements in the targeting antibody, changes in payload have been investigated as approaches to improving ADC efficacy. Efficacy with tubulin-binding agents such as MMAE is generally thought to be restricted to rapidly dividing cells, though some evidence supports activity on non- or slowly dividing cells.^11–13^ Development of ADC resistance in preclinical models has been broadly attributed to target downregulation, efflux pump induction, changes in ADC trafficking, and/or alterations in associated signaling pathways.^14^ For MMAE in particular, induction of the efflux pump Pgp has been correlated with loss of preclinical efficacy.^15,16^ In an attempt to overcome these liabilities, DNA-damaging agents, including pyrrolobenzodiazepine (PBD) dimers and *seco-*CBI dimers, have been explored as alternatives to microtubule inhibitors.^17–19^ Alkylation of DNA can lead to efficacy in both dividing and non-dividing cells, and subsets of these agents have been engineered to resist recognition by Pgp.^20^ Collectively, these data speak to the potential of alternative payloads driving durable responses with ADCs.

Here we describe the discovery, optimization, and characterization of Ly6E-targeted ADCs for cancer therapy. We show that a *seco-*CBI dimer (compound **2**) potently kills multiple cell lines, including lines not substantially affected by MMAE, and cells overexpressing Pgp with minimal loss of efficacy. The anti-Ly6E-CBI conjugate shows substantial inhibition of xenograft growth after a single dose, and in contrast to MMAE conjugates, repeated doses resulting in partial tumor regression do not lead to substantial tumor outgrowth. However, toxicity is apparent at these doses. A new Ly6E-targeted antibody generated by immunizing rats with Ly6E-containing VLPs (anti-Ly6Ev2) shows vastly improved binding, increased amounts internalized, and, when conjugated to cytotoxics, efficacy at substantially lower doses than the previous clinical antibody. Collectively these data describe investigations into the development of anti-Ly6E ADC therapeutic candidates.

## Results

### Characterization of seco-CBI-dimers and their conjugates

**Figure 1.**
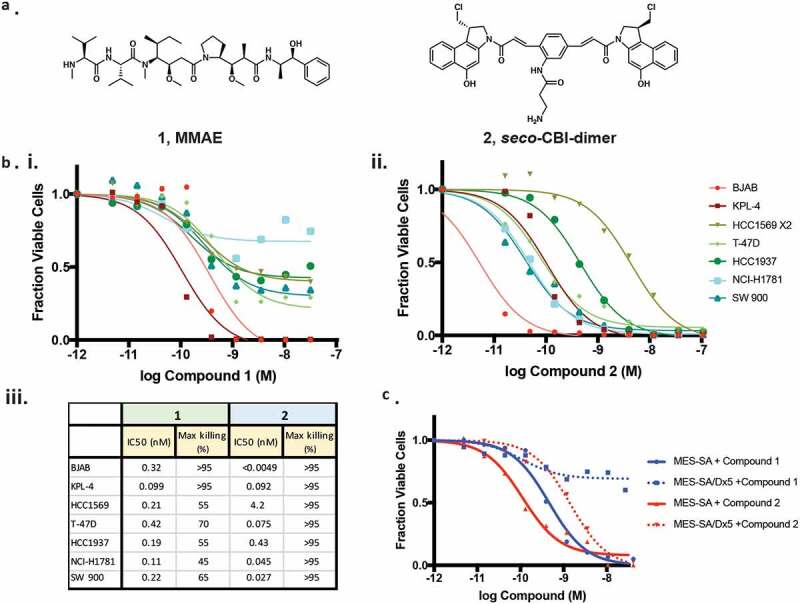
Characterization of cytotoxic small molecules. (*a*) Structures of MMAE (**1**) and a *seco*-CBI-dimer (**2**). (b) The indicated cell lines were grown in the presence of MMAE (*i*.) or **2** (*ii*.) for 3 d and the fraction of viable cells were detected using CellTiter Glo (Promega). Calculated potency and efficacy values are shown (*iii*.). (*c*) The impact of MMAE and **2** on the growth of MES-SA or MES-SA/DX-5 cells overexpressing Pgp was assessed as described in *B.*

In seeking an alternative to MMAE (compound **1**), we chose to evaluate the activity of compound **2**, a *seco*-CBI-dimer containing two reactive moieties capable of cross-linking DNA (Figure 1a).^19^ Incubation of either compound 2 or MMAE with multiple cell lines results in substantial cell death, though in all cases 2 was more potent (Figure 1b). Additionally, treatment with compound 2 resulted in >95% elimination of all cell lines while MMAE drove equal extents of killing in only two of the seven lines. We assessed the extent to which these drugs might evade resistance mechanisms by incubating them with MES-SA/Dx5, a multi-drug resistant cell line overexpressing Pgp, along with the parental MES-SA with low Pgp expression (Figure 1c).^21^ While both compounds kill >95% of the MES-SA cells in culture, MMAE kills only ~30% of the MES-SA/Dx5, whereas compound 2 shows only a modest loss of potency.

**Figure 2.**
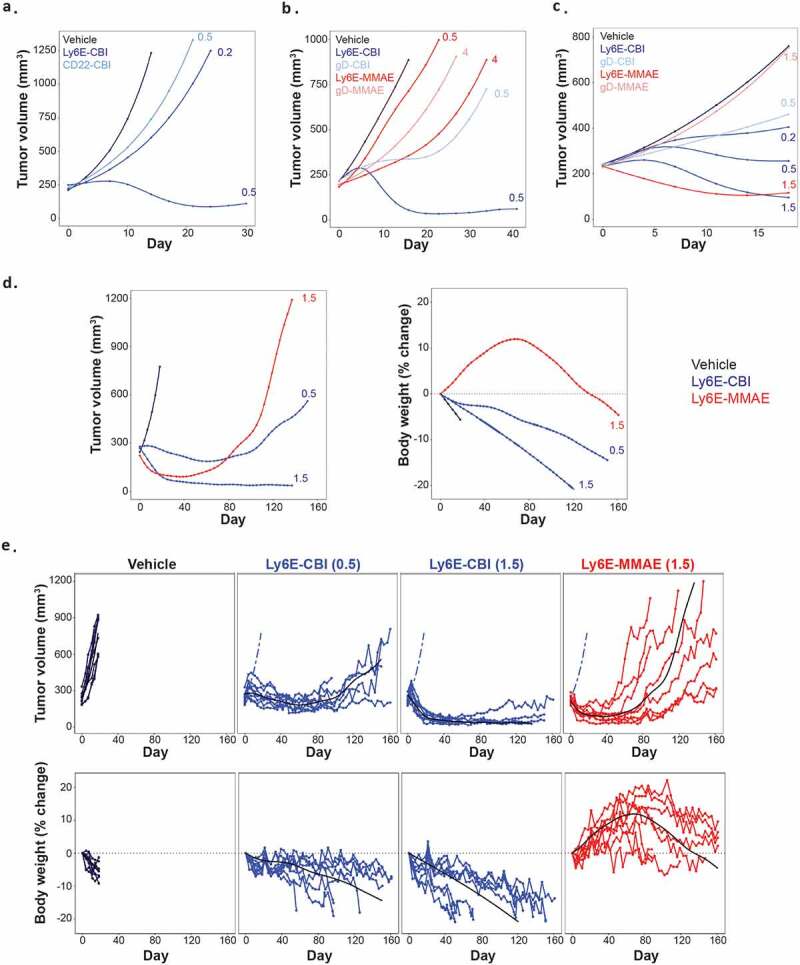
In vivo efficacy of anti-Ly6E ADCs in tumor xenograft models. (*a-c*) Growth of breast models HCC1569X2 (*a*) BR-05-028 (*b*) and lung cancer model SW900 (*c*) was assessed following a single administration of ADC. Durable efficacy of the ADCs was assessed with the SW900 model (*d-e*). Conjugates were administered every three weeks starting at day 0, and average (*d*) or individual (*e*) tumor volumes and body weight changes are plotted. Numbers next to traces indicate dose of each conjugate (in mg/kg) that was intravenously administered once at day 0. Cubic spline fitted tumor volumes are plotted for each treatment group (n = 5–7/group in *A-C* and n = 8 *D-E*). Cubic spline fits for vehicle group (dashed blue line) and treatment group (black line) are included for reference in *E.*

Following the characterization of the free compounds, we sought to generate anti-Ly6E-CBI ADCs. As previously described,^19^ the phenol moieties of 2 were masked by phosphates to improve solubility and enable conjugation, and the resulting prodrug was attached to a protease cleavable linker containing a maleimide (Supplementary Figure 1a). Site-specific conjugation to light chain (LC) K149C^22^ resulted in a homogenous ADC with a drug-to-antibody ratio (DAR) of two. Stability analysis of the ADC in both phosphate-buffered saline (PBS) and mouse whole blood showed no deconjugation of drug over 24 h at 37°C (Supplementary Figure 1b), though there was evidence of hydrolysis of the phosphate residues. The ADC was evaluated in four breast cancer models, including a xenograft model (HCC1569x2, IHC 3+, Figure 2a), and three patient-derived xenograft (PDX) models (BR-05-28 IHC 3+, Figure 2b; BR-05-14E, IHC 3+ and HBCx8, IHC 1+, both Supplementary Figure 2). As reported previously,^19^ *seco*-CBI ADCs are modified *in vivo* both by glutathione and alpha-1-microglobulin. On anti-Ly6E, the modification results in reduced tumor accumulation, potentially due to reduced anti-Ly6E affinity and/or increased size reducing ADC diffusion.^23^ Single doses of anti-Ly6E-CBI drove targeted activity across a range of Ly6E expression levels. Similar results were observed in lung cancer xenograft models (SW900, IHC 1/2+, Figure 2c, and NCI-H1781, IHC 1+, Supplementary Figure 2). Anti-Ly6E-CBI was generally more potent than anti-Ly6E-MMAE, but the non-targeted controls (anti-gD-CBI or anti-CD22-CBI) also showed more nonspecific activity than the corresponding MMAE conjugates (Figure 2c and Supplementary Figure 2). This off-target activity was more apparent in models with low Ly6E expression (Figure 2c and Supplementary Figure 2). Given the single-dose efficacy, we chose to explore the durability of anti-Ly6E-CBI and -MMAE in an SW900 xenograft model. Once tumors reached ~180 mm^3^, mice were treated with 0.5 or 1.5 mg/kg of anti-Ly6E-CBI or 1.5 mg/kg of anti-Ly6E-MMAE intravenously every 3 weeks up to eight times or until the mice were sacrificed due to body weight loss or overwhelming tumor burden (>1000 mm^3^). Consistent with the single-dose results, initial doses drove substantial tumor regression for both 1.5 mg/kg groups and more modest regression with 0.5 mg/kg of anti-Ly6E-CBI. Interestingly, the strong initial tumor control seen with the MMAE conjugate diminished over time (Figure 2d), and by the fifth dose (day 84) the improved efficacy seen versus the 0.5 mg/kg dose of anti-Ly6E-CBI was lost. Loss of efficacy was more apparent when tracking tumor volumes in individual mice (Figure 2e) where four-eighths of tumors completely escaped MMAE-ADC treatment control and mice needed to be sacrificed prior to the end of the study. By contrast, no mice were sacrificed due to tumor size for either of the *seco-*CBI groups over more than 5 months of treatment. This durable efficacy even at sub-optimal doses is contrasted by the comparatively poor tolerability of conjugates derived from 2. Mice treated with anti-Ly6E-MMAE showed initial weight gains prior to the tumors beginning to rebound (Figure 2d). By contrast, mice treated with anti-Ly6E-CBI showed moderate or substantial weight loss at 0.5 and 1.5 mg/kg, respectively, and a total of seven animals across the two groups were sacrificed due to loss of >20% of their body weight (Figure 2e) prior to the end of the study.

### Identification of improved anti-Ly6E antibodies

**Figure 3.**
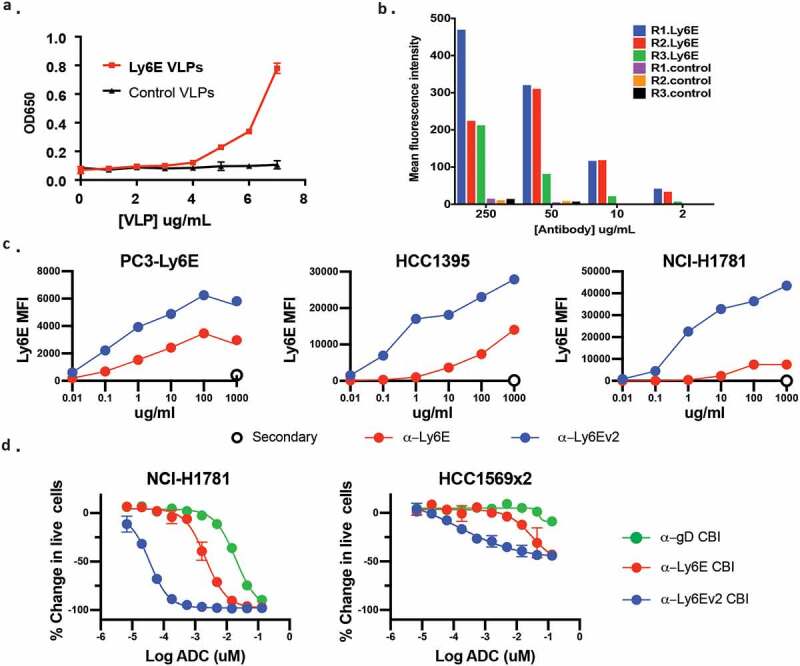
Discovery and in vitro characterization of anti-Ly6Ev2. (*a*) Viral-like particles from HEK293T cells (±) Ly6E were assessed by ELISA for the presence of the antigen. (*b*) Polyclonal antibodies were isolated from rats before (control) and after immunization with Ly6E-VLPs. Binding of the polyclonal antibodies to PC3 cells overexpressing Ly6E was assessed by FACS. (*c*) Flow cytometry analysis of anti-Ly6E and anti-Ly6Ev2 with three cell lines validated to express Ly6E by Western blot. Points represent the average of two replicate samples. (*d*) Anti-Ly6E, anti-Ly6Ev2, or control (gD) *seco-*CBI conjugates were incubated with the indicated cells lines for 5 d and the number of live cells was assessed using CellTiter-Glo

Though encouraged by the increased durability observed using the *seco-*CBI conjugates, including at a dose showing limited regression, we sought ways to mitigate the observed toxicity while maintaining efficient tumor control. We hypothesized that antibodies with higher affinity and/or more efficient internalization might deliver larger doses of drug to the tumors while maintaining tolerable exposure to healthy tissues. With this in mind, we endeavored to discover additional anti-Ly6E antibodies. In previous antibody campaigns,^1^ immunizing rats with recombinant Ly6E yielded only a single clone with detectable binding to Ly6E-expressing cells, anti-Ly6E. We hypothesized this poor response was caused by misfolding of the antigen as evidenced by substantial laddering in non-reducing SDS-PAGE gels (data not shown). Repeated attempts to generate high-quality antigen for immunizations failed to realize significant improvements. Parallel attempts to immunize rats with DNA to enable expression of huLy6E in rat cells provided four positive clones, all with worse binding to overexpressed Ly6E than the previous clinical lead (data not shown). As a final approach, we investigated the immunization of rats with Ly6E-containing VLPs. Co-expression of Ly6E with genes driving viral budding results in production of vesicles that can be isolated and characterized prior to immunization. Maintenance of proteins within their native membrane-bound state helps stabilize the extracellular domains in a native conformation, and the high surface area to volume ratio of the VLPs minimizes contamination from intracellular protein and helps to focus the immune response on the overexpressed protein. Enzyme-linked immunosorbent assay (ELISA) analysis of VLPs containing Ly6E showed marked integration of the protein (Figure 3a), and quantitative Western blotting allowed us to estimate that Ly6E comprised ~0.5% of the total protein (Supplementary Figure 3). Polyclonal antibodies isolated from rats before and after immunization with Ly6E-VLPs or VLPs alone were incubated with PC-3 cells overexpressing Ly6E prior to analysis by flow cytometry. Across a range of polyclonal concentrations, all three rats immunized with Ly6E-containing VLPs showed substantially higher binding than control rats (Figure 3b). Thirteen Ly6E-ELISA positive hybridomas were recovered from the immunized animals, but only the top binding clone showed substantial binding to Ly6E over-expressing cells (data not shown). This clone was humanized (anti-Ly6Ev2) without apparent loss of binding (Supplementary Figure 4a).

While biophysical characterization of binding was precluded by the poor quality of antigen, alternative approaches were undertaken to characterize anti-Ly6Ev2. Direct binding of antigen-binding fragment (Fab)-luciferase fusions^24^ to Ly6E-expressing NCI-H1781 cells was assessed following a 4 h incubation at 4°C (Supplementary Figure 4b). The anti-Ly6Ev2 fusion bound with an apparent EC50 of 7 nM, while anti-Ly6E fusion did not saturate, and at the maximum tested concentration (200 nM) showed ~2% of maximal signal shown by anti-Ly6Ev2. Competitive binding experiments were undertaken to account for potential interference by the fused luciferase (Supplementary Figure 4c). Under this set of conditions, anti-Ly6Ev2 showed an affinity of 9 nM, whereas no substantial competition was observed at up to 200 nM of Fab for anti-Ly6E. Polyspecificity was analyzed by examining binding to baculovirus particle-coated ELISA plates.^25^ The score of 0.17 is well below the cutoff for positivity (1.0), suggesting the anti-Ly6Ev2 binds specifically to Ly6E. While no detailed mapping was performed, N-terminal tags severely affected monovalent binding of the Fab in cell-based assays, suggesting the N-terminus may be part of the binding epitope.

Given the mixture of antigens administered in VLPs, additional steps were taken to validate the specificity of the new clone. We compared cell binding of anti-Ly6E and anti-Ly6Ev2 across a range of cells using Western blotting analysis with an alternative antibody as an orthogonal validation for true expression (Figure 3c and Supplementary Figure 5). Anti-Ly6Ev2 showed substantially enhanced binding to five cell lines expressing endogenous Ly6E by immunoblot, while on immunoblot-negative cells, both the original and new antibody showed similar background staining. A format-matched *seco-*CBI-ADC was generated from anti-Ly6Ev2, and its cell killing was evaluated in lung and breast tumor-derived cell lines (Figure 3d). In each case, anti-Ly6Ev2-CBI showed an ~100-fold increase in potency vs. anti-Ly6E-CBI and ~1000-fold increase vs. the untargeted control.

**Figure 4.**
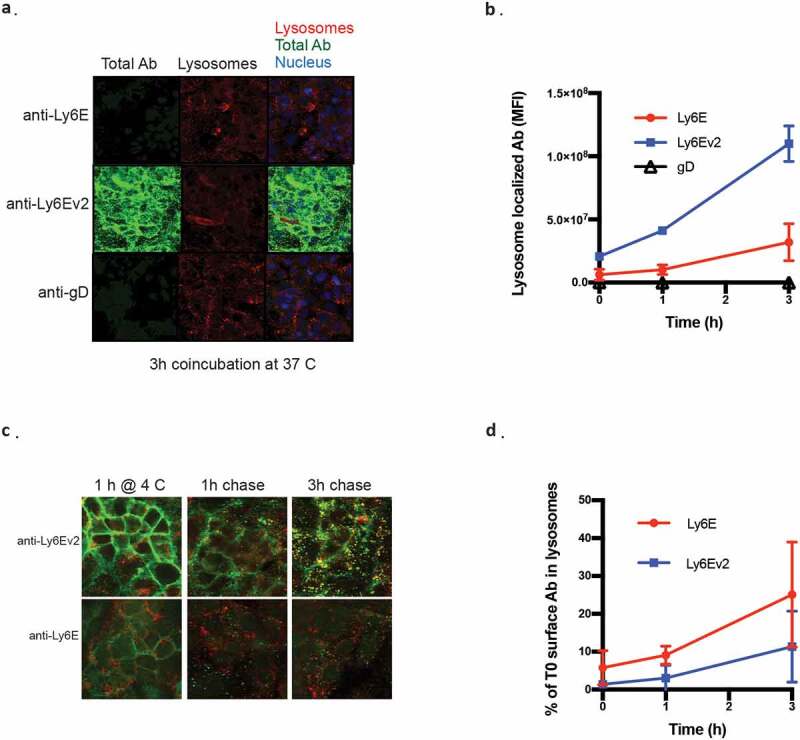
Fluorescent microscopy characterization of anti-Ly6E antibodies. *(a)* Kuramochi cells were incubated with 2 µg/ml of the indicated antibodies at 37°C for the indicated periods of time and stained with donkey anti-human IgG-488 (green), donkey anti-rabbit IgG AF546 for lysosomes (red), and Hoechst DNA staining for nuclei (blue). Colocalization of hIgG with lysosomes is shown in yellow. *(b)* Quantification of lysosome-localized antibody over time. *(c)* Kuramochi cells were incubated with 2 µg/ml of the indicated antibodies at 4°C for 1 h, washed, and then incubated at 37°C for the indicated periods of time. Staining is as described in *A. (d)* Quantification of internalized antibody in the pulse chase normalized to total antibody seen at t = 0 h

We assessed binding and internalization of the two antibodies by fluorescent microscopy to better understand the origin of the enhanced efficacy. Kuramochi cells, an ovarian cell line model, were used as the model system due to their Ly6E expression and cellular morphology amenable to high throughput imaging. When the cells are continuously exposed to 2 µg/ml of the antibodies for 3 h, the greatly enhanced binding of anti-Ly6Ev2 results in substantially increased amounts of antibody both on the surface and colocalized with the lysosomal marker. Interestingly, while the cell surface staining of anti-Ly6Ev2 was more than 20-fold greater than was seen for anti-Ly6E (Supplementary Figure 6a), the lysosome-localized antibody was only about 3-fold higher (Figure 4b). We considered two potential drivers for this gap: 1) the antibodies might have different internalization rates, or 2) antibody degradation in the lysosome might reduce the amount of detected antibody. To assess the antibodies’ internalization rates, we performed a pulse-chase experiment where antibodies were incubated with cells for 1 h at 4°C, washed, and internalization was monitored over time. While the total amount of internalized anti-Ly6Ev2 remained higher in this experimental paradigm (Supplementary Figure 6b), the fraction of bound antibody bound at t = 0 that internalized within our 3 h window was ~2-fold higher for anti-Ly6E than for anti-Ly6Ev2 (Figure 4d). These data suggest that surface-bound anti-Ly6E internalizes somewhat more efficiently than the second-generation antibody, and the observed improvements in *in vitro* killing are driven primarily by enhanced binding.

### In vivo behavior of anti-Ly6Ev2

**Figure 5.**
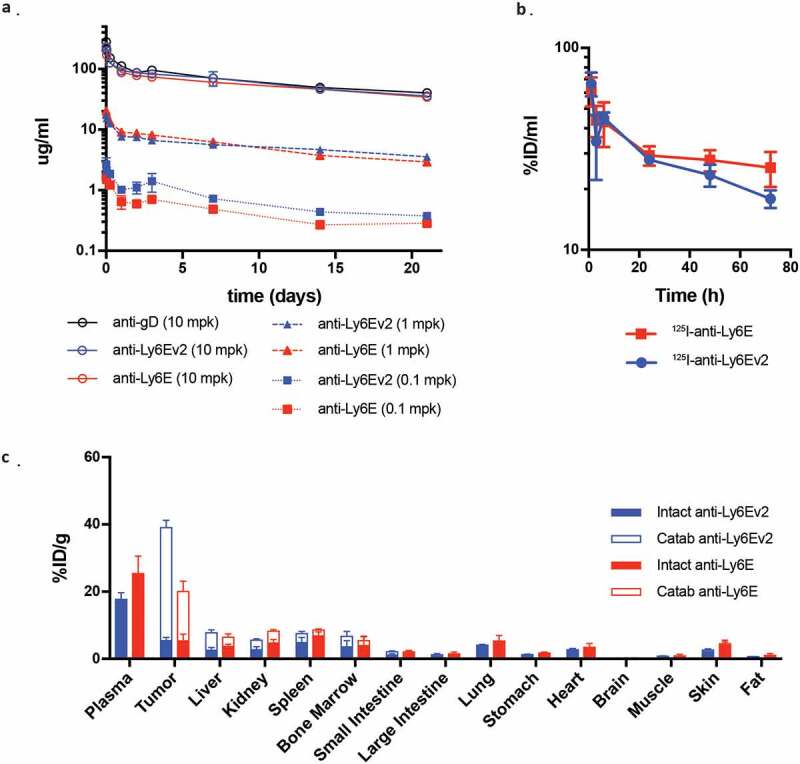
Pharmacokinetics and biodistribution of LY6E targeting antibodies. (*a)* Serum time-activity curves for anti-Ly6Ev2 (blue) and anti-Ly6E (red) in a dose-ranging pharmacokinetic study in non-tumor bearing female SCID.bg mice demonstrate linearity across 0.1, 1 and 10 mg/kg doses and comparable clearance to a non-binding control antibody (anti-gD) at 10 mg/kg. (*b-c*) HCC1569x2 tumor-bearing mice received a single intravenous bolus of radiolabeled Ly6E targeting antibodies (1 mg/kg), anti-Ly6E (red) or anti-Ly6Ev2 (blue). (*b*) Mice were bled between 1 h and 72 h post injection and intact antibody signal in whole blood plotted to characterize the systemic exposure during the course of the biodistribution study. (c) Tissue distribution at 72 h, with values are reported as a percentage of injected radioactive dose normalized to gram of dry blotted tissue (%ID/g) to convey changes in enrichment. Filled bars represent intact antibody values, while hollow bars represent catabolized values. The total value of the stacked bars represents intact and catabolized exposure up to that time point. All graphs are mean SD for each group with n = 4

We next sought to assess *in vivo* pharmacokinetic behavior of anti-Ly6Ev2. SCID mice were administered 0.1 to 10 mg/kg of either anti-Ly6E antibody or an untargeted control, and serum antibody levels were assessed by ELISA (Figure 5a). The antibodies showed dose-proportional exposure with no evidence of increased clearance versus the control (Supplementary Table 1). We further investigated distribution of the two anti-Ly6E antibodies in mice implanted with HCC1569x2 tumors. The anti-Ly6E antibodies were simultaneously labeled with ^125^I, a modification attached primarily to tyrosine that will rapidly diffuse away following antibody catabolism, and with a DOTA-chelated ^111^In, which accumulates in the catabolizing cell.^26^ This pair of labels enables simultaneous quantitation of tissue distributions for both intact and degraded antibodies. Seventy-two hours following a 1 mg/kg injection, most tissues showed similar levels of both intact and catabolized antibodies, with overall low levels of catabolism (Figure 5c). Within the tumor, anti-Ly6Ev2 showed ~2.5-fold more catabolized antibody than anti-Ly6E, consistent with efficient antigen-specific targeting. Interestingly, in contrast to what had been seen in non-tumor bearing mice, plasma levels of anti-Ly6Ev2 started to diverge at 24 h and were substantially below anti-Ly6E after 3 d (Figure 5b). Similar but less dramatic differences were apparent within 24 h (Supplemental Figure 7). This differential exposure in tumor-bearing mice is indicative of target-mediated drug disposition (TMDD), a hypothesis that is further supported by the increase in tumor-localized catabolized antibody for anti-Ly6Ev2 (Figure 5c). While TMDD adds some complexity to the dose–efficacy relationship, rapid internalization of the drug into tumors may both enhance targeted efficacy and minimize systemic exposure, thus preventing off-target toxicities.

**Figure 6.**
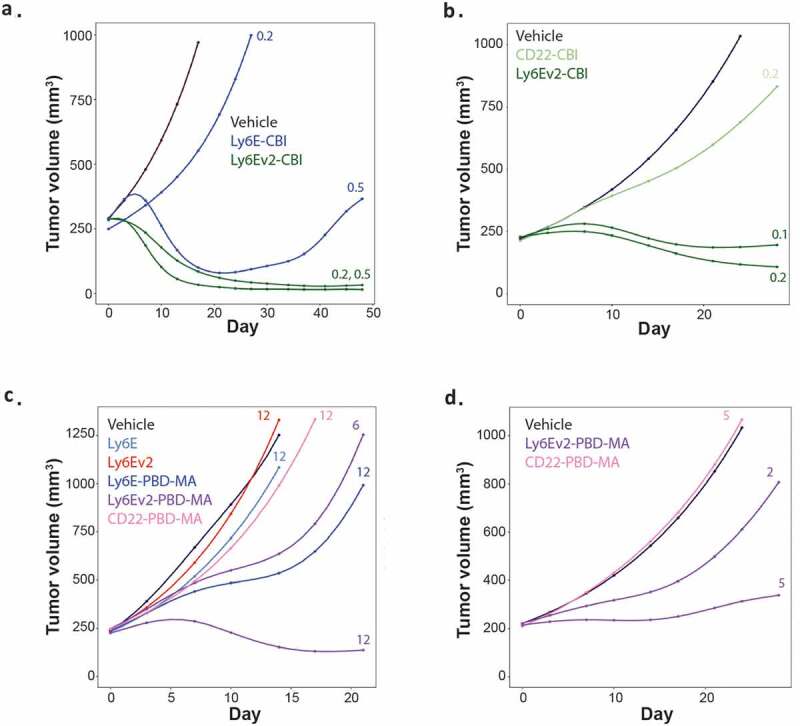
*In vivo* efficacy of anti-Ly6Ev2-CBI and anti-Ly6Ev2-PBD-amide in tumor xenograft models. (a, c) HCC1569X2 breast cancer, and (b, d) SW900 lung cancer. Numbers next to traces indicate dose of each conjugate (in mg/kg) that was intravenously administered once at day 0. Cubic spline fitted tumor volumes are plotted for each treatment group (n = 5–8/group)

The effect of the enhanced antibody on ADC efficacy was evaluated in both breast and lung cancer xenograft models (HCC1569x2 and SW900). In the HCC1569x2 model (Figure 6a), a single dose of anti-Ly6Ev2-CBI at 0.2 mg/kg provided complete tumor control out to 7 weeks, whereas conjugates of the previous clinical antibody showed almost no tumor control at 0.2 mg/kg, and at 0.5 mg/kg showed limited regression followed by rebound beginning around 3 weeks. In the SW900 model, doses as low as 0.1 mg/kg of anti-Ly6Ev2-CBI enabled modest tumor regression, and the 0.2 mg/kg dose was substantially separated from the untargeted control (anti-CD22) (Figure 6b). Though direct comparisons are challenging between compounds and doses due to differential tumor burdens, essentially no body weight loss was observed for anti-Ly6Ev2-CBI at 0.1 or 0.2 mg/kg after 21 d of treatment (Supplemental Figure 8a-b). The potent efficacy observed in the SW900 model is in contrast to a previous experiment with anti-Ly6E-CBI (Figure 2e) where 1.5 mg/kg of ADC was required to drive regressions and substantial tumor growth inhibition was observed with 0.5 mg/kg of an untargeted agent. Encouraged by this preclinical efficacy, we explored whether the improved efficacy would extend to less potent payloads where off-target activities might be less of a concern. Conjugates were made to a pyrrolobenzodiazepine dimer mono-amide (PBD-MA) (Supplementary Figure 9). In this cytotoxic agent, one of the two DNA reactive sites is inactivated, resulting in a compound that alkylates rather than cross-links DNA. As a consequence, PBD-MA conjugates show reduced toxicity to both healthy and malignant cells. PBD-MA conjugates were assessed in the HCC1569x2 xenograft model using single doses of the therapeutic agents (Figure 6c). Anti-Ly6Ev2-PBD-MA showed regression at 12 mg/kg and partial tumor growth inhibition at 6 mg/kg. The activity of a 12 mg/kg dose of anti-Ly6E-PBD-MA closely resembled the 6 mg/kg dose of the second-generation antibody. This efficacy extended to the SW900 model where 5 mg/kg was nearly sufficient to drive tumor stasis. In both cases, equivalent doses of conjugates targeted to a control antigen showed no impact on tumor growth (Figure 6d) and minimal impact was seen on body weight (Supplementary Figure 8c-d).

## Discussion

Targeted delivery of cytotoxics is a clinically validated approach to treat malignancy. To date, nine ADCs have been approved by the Food and Drug Administration, including ones that deliver microtubule inhibitors, topoisomerase inhibitors, and DNA-damaging agents.^27^ While ADCs are clinically effective, the median duration of response for approved agents is often 6–12 months (e.g., brentuximab vedotin (Adcetris®) in Hodgkin’s lymphoma at 6.7 months,^28^ trastuzumab emtansine (Kadcyla®) in HER2+ breast cancer at 12.6 months,^29^ inotuzumab ozogamicin (BESPONSA®) in acute lymphoblastic leukemia at 4.6 months,^30^ and enfortumab vedotin (Padcev®) in urothelial cancer at 7.6 months).^31^ This may reflect an optimistic assessment of ADCs as a field, as poor durability can be a factor in choosing not to advance agents with partial activity, e.g., DLYE5953A.^7^ The origins of this poor durability are complex. *In vitro* studies have identified drug efflux pumps that reduce the efficacy of some of these cytotoxics (e.g., Pgp, ABCG2) and clinical efficacy of both gemtuzumab ozogamicin (Mylotarg®) and inotuzumab ozogamicin have been linked to lower expression of Pgp.^14,32,33^ In the Phase 1 trial with anti-Ly6E-MMAE, the three patient biopsies tested post-progression all showed increases in Pgp levels.^7^ Antigen loss has been repeatedly identified preclinically as a means of evading ADC-mediated killing, though studies investigating the clinical relevance of this mechanism on brentuximab vedotin have found mixed results.^34,35^ Notably, in the limited number of patients investigated for Ly6E expression following progression, no antigen loss was observed.^7^ Multiple additional mechanisms of resistance have been described to occur *in vitro*, including heterogeneity in tumor growth rate or adaptive signaling pathways resulting in incomplete efficacy, and changes in ADC internalization, linker cleavage, or drug import from the lysosome driving reduced exposure to the cytotoxic agent.^14^ To the best of our knowledge, these mechanisms have not been observed in patients. The goal of this work has been the development of a second-generation anti-Ly6E ADC that overcomes the observed limitations of DLYE5953A.

We chose to focus on a *seco*-CBI-dimer as the payload, a molecule that is intended to cross-link DNA and evade some of the emergent resistance observed with both microtubule inhibitors and DNA mono-alkylators. A small number of ADCs with related payloads have been evaluated in the clinic, including MDX-1203^36^ and SYD985 (trastuzumab duocarmazine),^37^ though each delivers a cytotoxic molecule that mono-alkylates (rather than cross-links) DNA. Both agents showed reasonable tolerability in Phase 1 trials, and the Phase 2 trial of SYD985 is ongoing. In our hands, the unconjugated *seco*-CBI-dimer **2** shows potent and complete efficacy across a range of cell lines, regardless of proliferation rate, and in the presence of overexpressed Pgp (Figure 1), validating the approach as a means to avoid resistance. When conjugated to Ly6E-targeted antibodies, 2 drives enhanced and durable efficacy over more than 5 months, even at doses resulting in tumor stasis rather than regression (Figure 3a). These results are in stark contrast to what was observed with MMAE-derived ADCs, suggesting that *seco-*CBI conjugates may result in more durable therapies. A recent report^23^ shows that anti-Ly6E-CBI is subject to modification by A1M, resulting in reductions in target binding, *in vitro* potency, and accumulation in tumors. Despite also being modified by A1M in whole blood, anti-Ly6Ev2-CBI shows efficacy at doses as low as 0.1 mg/kg. We speculate that differences in epitope and/or affinity enable anti-Ly6Ev2-CBI to continue to bind Ly6E in spite of A1M additions.

As is always the case for ADCs, therapeutic efficacy must be weighed versus tolerability. Mice treated with therapeutically active doses of anti-Ly6E-CBI showed substantial weight loss, and a subset of mice treated with multiple doses did not survive the regimen (Figure 2e). Anti-Ly6E (and anti-Ly6Ev2) shows minimal murine cross-reactivity (data not shown), suggesting that these effects are likely due to nonspecific uptake of the ADC. This can potentially be resolved using anti-Ly6Ev2-conjugates that drive efficacy at reduced doses, but the potential risk of Ly6E-targeted toxicity is increased. Of note, in a Phase 1 trial with anti-Ly6E-MMAE, the maximum tolerated dose of 2.4 mg/kg^7^ is the same as or higher than the approved dose of MMAE ADCs (1.8 mg/kg brentuximab vedotin,^38^ 1.25 mg/kg enfortumab vedotin,^39^ and 2.4 mg/kg polatuzumab vedotin)^40^ and the nature of the toxicities is broadly similar, including peripheral neuropathy, neutropenia, and gastrointestinal toxicities. Antigen-specific effects appeared to be largely limited to alopecia and immune-related reactions, responses that are not the source of the dose-limiting toxicity.^7^ This is consistent with other observations in the field, where ADC toxicity is typically driven by payload rather than by antigen. Two duocarmycin analog conjugates, SYD985 and MDX-1203, have been evaluated in clinical trials.^36,37^ Relatively few dose-limiting toxicities have been observed for either drug, but these lack the second reactive moiety present in **2**. SYD985 is currently in a Phase 3 trial (NCT03262935), whereas MDX-1203 clinical development has stopped due to limited efficacy at 8 and 15 mg/kg. Alternative ADCs containing DNA cross-linking agents, most notably rovalpituzumab tesirine,^41^ have shown unacceptable clinical profiles, and as a consequence, clinical development has been stopped. In light of this risk, we also investigated activity with an alternative and less potent class of cytotoxic payload.

The use of less potent targeted cytotoxics, including ADCs bearing PBD-mono alkylators (e.g., DGN462)^42^ and topoisomerase inhibitors,^43^ has recently been explored in the clinic as a means to drive therapeutic efficacy while attempting to avoid the most common and significant toxicities. Higher doses of these ADCs are more likely to achieve linear pharmacokinetics and more consistent exposure. Furthermore, the higher doses may increase target saturation on perivascular tumor cells, resulting in more efficient tumor-penetration and avoiding sub-efficacious dosing at the center of the solid tumor.^44^ Encouragingly, the improved efficacy of anti-Ly6Ev2-CBI, which appears to be driven by the increased binding of the antibody, also extends to the PBD-MA conjugates. While previous work suggests that high-affinity antibodies may inhibit effective tumor penetration,^45^ these studies have typically focused on antibodies with low-to-sub-nanomolar affinities. We believe we were able to enhance tumor accumulation without limiting radial penetration from the vasculature due to the very weak monovalent affinity of anti-Ly6E. Demonstration that the same improved binding results in increased efficacy for conjugates with a 50-fold difference in potency suggest anti-Ly6Ev2 may extend the gamut of Ly6E-targeted therapies.

In summary, we have described efforts to maximize the efficacy and durability of the anti-tumor response for Ly6E-targeted ADCs. We have demonstrated that a *seco*-CBI-dimer drives potent tumoricidal activities across a swath of cell lines and that it maintains activity in cells resistant to MMAE. This in turn translates to durable efficacy in multi-dose xenograft studies. The use of a VLP-immunization technique enabled the discovery of a tighter binding antibody with greater total internalization, which enables the generation of ADCs showing increased potency and reduced systemic exposure due to TMDD. Collectively, we believe this work provides potential alternatives to the clinically active anti-Ly6E ADC, DLYE5953A.

## Materials and methods

### DNA and protein design and production

All antibodies in this work are numbered using EU numbering systems for constant domains.^46^ Antibody constructs were generated by gene synthesis (GeneWiz) or through mutagenesis using the Q5 Site-Directed Mutagenesis Kit (New England Biolabs, E0554S). Chimeric antibodies were humanized using the CDR-graft methodology introducing murine Vernier residues to optimize affinity.^47^ Recombinant antibodies, including those containing cysteine mutations, were produced by transient transfection of Chinese hamster ovary (CHO) cells with recombinant DNA and purified by affinity chromatography.

### VLP generation and characterization

Expi293 cells were co-transfected with a mammalian expression construct encoding the full-length Ly6E and a mammalian expression construct encoding for MLGag.^48^ Seven days post-transfection, VLPs were purified from the supernatant using ultracentrifugation as previously described.^49^ VLP concentrations were measured using a Bradford assay. Incorporation of Ly6E was confirmed by ELISA. Nunc Maxisorp ELISA plates (Thermo Scientific, 44–2404-21) were coated with various concentrations of VLPs diluted in coating buffer (50 mM carbonate, pH 9.6) at 4°C overnight. The plates were washed with wash buffer (1xPBS with 0.05% Tween20) and then blocked with ELISA assay diluent (PBS/0.5% bovine serum albumin (BSA)/0.05% polysorbate 20) at room temperature (RT) for 1 h. Plates were then incubated at RT for 1 h with anti-Ly6E (9B12; 1 μg/ml diluted in ELISA assay diluent). The plates were washed with wash buffer and the bound antibody was detected with goat anti-rat horse-radish peroxidase secondary antibody (Jackson Immunol Lab, 112–035-003). The plates were incubated at RT for 30 minutes, washed with wash buffer and developed with TMB solution (Surmodics, USA, TMBS-0100-01). Plates were read at 630 nm.

### VLP immunization

Animals used in these studies were maintained in an Association for Assessment and Accreditation of Laboratory Animal Care (AAALAC)-accredited animal facility. All experiments were performed in compliance with Genentech’s Institutional Animal Care and Use Committee (IACUC) and National Institutes of Health’s Office of Laboratory Animal Welfare Guidelines. Approval of the study design was obtained from the Genentech IACUC prior to the start of this work.

Sprague Dawley rats (Charles River Laboratories, Hollister, CA) were immunized with Ly6E VLPs in PBS along with Ribi adjuvant (Sigma). The rats were then boosted three times with additional Ly6E VLPs, every 2 weeks. This was followed by four injections of a plasmid encoding for Ly6E cDNA via Genegun over 2 weeks. DNA/gold particle bullets are prepared essentially as previously described.^50,51^ Each bullet for DNA was prepared to contain a total of 1 μg of DNA coated onto 0.5 mg of gold particles (BioRad, 1652264). Bullets were stored at 4°C in the dark in the presence of desiccant pellets.

Polyclonal antibodies were purified by Protein A and assayed by fluorescence-activated cell sorting (FACS) as described below. To generate monoclonal antibodies, hybridoma fusions were performed as previously described except with a myeloma partner SP2ab that enables the surface display of IgG cell.^52,53^ Hybridoma supernatants were harvested and IgG was purified from supernatants using MabSelect SuRe (GE Healthcare, Piscataway, NJ, USA, 17543803). Anti-Ly6E hybridomas were identified by ELISA and FACS screening. The variable light chain and variable heavy chain sequences of anti-Ly6E hybridoma were determined using 5ʹ RACE followed by sequencing of the PCR-amplified products.

### Anti-Ly6E screening

Control PC3 and Ly6E-transfected PC3 cells were stained with various concentrations of polyclonal IgG purified from Ly6E VLP-immunized rat sera, or hybridoma supernatant-purified IgG at 4°C for 30 minutes. Cells were washed twice with FACS buffer (PBS with 0.5% BSA and 2 mM EDTA) and stained with Alexa Fluor® 647 conjugated goat anti-rat IgG (Jackson ImmunoReaserch, 112–607-008). After washing twice with PBS, cells were resuspended in FACS buffer with propidium iodide (BD Bioscience) and run on a BD LSRFortessa™ X-20 cell analyzer and data analyses were performed using FlowJo v.9.7.7 software.

### Linker drug production

Linker drugs for MMAE- and *seco*-CBI-dimer-conjugates were prepared as previously described.^19,54^ Detailed synthetic procedures for the PBD-MA linker drug are provided in the Supplementary Methods.

### Production of ADCs

ADCs were made from antibodies containing the LC K149C mutation, termed ThioMabs, essentially as described.^55^ All conjugates were generated with less than 5% aggregate and at DAR >1.8.

### Cell-based binding

Cell lines were cultured as recommended by the ATCC. Prior to staining, adherent lines were released by Accutase treatment, all cells were filtered and equilibrated in BD Stain buffer (BSA). Cells were stained with primary antibodies at indicated concentrations for 1 h at 4°C, washed x 3, stained with secondary antibody (Jackson ImmunoResearch 109–606-003) (1:100) for 1 h at 4°C, washed, stained with Fixable Viability Dye eFluor 780 (eBioscience, 65–0865-14) (1:1000) for 1 h at 4°C, and washed x 2. Samples were fixed in PBS with 1% paraformaldehyde (PFA) prior to analysis. Samples were assessed on a BD LSRFortessa. Following the exclusion of dead cells, singlets were assessed for antibody binding using FlowJo software.

For equilibrium binding experiments, cells were mixed with Fabs and incubated at the indicated concentrations for 4 h at 4°C, washed x 3, and resuspended in 20 μL of PBS. Luciferase substrate (Promega, 50 µL) was incubated with the cells prior to analysis on a luminometer.

### Polyspecificity assessment

Nonspecific binding of anti-Ly6Ev2 was determined using an ELISA to baculovirus-coated plates as described.^25^

### Immunoblot analysis

Approximately five million cells were lysed in RIPA Lysis and Extraction Buffer (ThermoFisher Scientific, #89900) according to the manufacturer’s recommendations. Soluble protein (20 µl) was separated by SDS-PAGE and transferred to PVDF membrane. The membrane was incubated with Odyssey blocking buffer (LI-COR, 927–50010), stained with 2 µg/ml of GEN-93-8-1^1^ in blocking buffer, washed x 4, probed with Goat anti-Rabbit IgG H&L (IRDye® 800CW) pre-adsorbed (Abcam, ab216773) and washed x 3 prior to analysis on an LI-COR Odyssey.

### Microscopy and quantitation

On Day 0, Kuramochi cells were seeded in 384-well Cell Carrier plates (Perkin Elmer) and allowed to adhere overnight to attain an 80% confluency. The next day, cells were treated with unconjugated antibodies at 2 and 20 ug/mL with cold media containing 10 ug/mL leupeptin and 5 uM pepstatin protease inhibitors. Antibodies were allowed to bind for 1 h on ice. Cells with pulse treatments were washed with cold media containing protease inhibitors, while cells with continuous treatment were left alone. Cells were incubated at 37°C, 5% humidified CO_2_ for 1 h and 3 h for antibody internalization. After incubation, cells were fixed with 4% PFA/4% sucrose in PBS for 10 min at RT, and then cells were washed six times with 1x PBS. Cells were blocked and permeabilize with 2% donkey serum containing 0.05% saponin (block buffer) for 1 h. Block buffer was removed, then rabbit anti-LAMP1 (Sigma Aldrich, L1418) primary antibody in block buffer was added and incubated overnight. The next day, cells were washed six times with PBS containing 0.05% saponin, and then secondary antibodies donkey anti-human IgG-488 (Jackson ImmunoResearch, 709–546-149) and donkey anti-rabbit IgG AF546 (Invitrogen, A10040) were added and incubated for 1 h at RT. Cells were washed 6 times with PBS containing 0.05% saponin. Hoechst DNA staining in PBS was added. Cells were imaged using an In Cell Analyzer 6000 with a 10x objective for Hoechst signal (Ex405 nm/Em415-475 nm), AF488 signal (Ex499nm/Em520nm) and AF546 signal (Ex552 nm/Em570-590 nm) and object mean fluorescence intensities determined (see Figure 4b).

### Whole blood stability of ADCs

Whole blood stability of ADCs was evaluated using protocols as previously described^56^ with the following modification: specific affinity capture was performed using an anti-Ly6E anti-idiotypic antibody developed in house conjugated to beads.

### ELISA assays for pharmacokinetic analysis

Total antibody concentrations in serum were determined with a Generic Total Antibody ELISA. Nunc® MaxiSorp™ 384-well plates (Thermo Fisher Scientific, Waltham, Massachusetts, USA) were coated with 25 μL/well of 0.8 μg/mL sheep anti-human IgG antibody (Binding Site, San Diego, CA, USA) diluted in coat buffer (0.05 M carbonate/bicarbonate buffer pH 9.6) and incubated overnight at 4°C. The plates were washed 3 times with wash buffer (0.5% Tween-20 in PBS buffer, pH 7.4) and treated with 50 μL/well block buffer (PBS/0.5% BSA/15 ppm Proclin, pH 7.4) for 1 to 2 h at RT. The plates were again washed three times with wash buffer, and then 25 μL/well of samples diluted in sample diluent (PBS/0.5% BSA/0.05% Tween 20/5 mM EDTA/0.25% CHAPS/0.35 M NaCl/15 ppm Proclin, pH 7.4) was added to the plate and incubated on a shaker for 2 h at RT. The plates were washed 6 times with wash buffer. A detection antibody, goat anti-human antibody-horseradish peroxidase (HRP) (Bethyl Laboratories, Inc., Montgomery, TX, USA, A80-119P), diluted to 100 ng/mL in assay buffer (PBS/0.5% BSA/15 ppm Proclin/0.05% Tween 20, pH7.4) was added to the plate at 25 μL/well and incubated on a shaker for 1 h at RT. The plates were washed 6 times with wash buffer and developed using 25 μL/well of TMB peroxidase substrate (Moss Inc., Pasadena, Maryland) for 15 minutes followed by 25 μL/well of 1 M phosphoric acid to stop the reaction. Absorbance was measured at 450 nm against a reference wavelength of 620 nm. The concentration of the samples was extrapolated from a 4-parameter fit of the standard curve. The reportable assay range was 0.156–10 ng/mL. Following a minimum dilution of 1:100, the lower limit of quantitation is 15.6 ng/mL.

### In vivo *efficacy*

The efficacy of Ly6E ADCs was investigated in a mouse xenograft model of HCC1569X2 (human breast cancer), SW900, NCI-H1781 (human lung cancer), or BR-05-028, BR-05-014E, HBCx8 (patient-derived breast cancer).

Human lung cancer cell lines SW900 and NCI-H1781 were obtained from ATCC (Manassas, VA). Human breast cancer HCC1569x2 cell line (generated at Genentech) was derived from parental HCC1569 (ATCC) with optimal growth in mice. Each cell line was authenticated by short tandem repeat (STR) profiling using the Promega PowerPlex 16 System and compared with external STR profiles of cell lines to determine cell line ancestry. Animal studies using these cell lines were carried out at Genentech in compliance with National Institutes of Health guidelines for the care and use of laboratory animals and were approved by the IACUC at Genentech. To establish the model, five million tumor cells (suspended in 0.1–0.2 mL of HBSS with Matrigel) were inoculated into the thoracic mammary fat pad (for breast model) or subcutaneously at the flank area (for lung model) of female C.B-17 SCID-beige mice (Charles River Laboratory; Hollister, CA).

Animal studies using PDX models of BR-05-014E and BR-05-028 were carried out at WuXi AppTec Co., Ltd. (Shanghai, China). All the procedures related to animal handling, care and the treatment in the study were performed according to the guidelines approved by the IACUC of Wuxi AppTec following the guidance of the AAALAC. These models were derived from surgically resected clinical samples and maintained through serial implantations in mice. Human origin of PDX tumors was confirmed by PCR testing. To set up the model for an efficacy study, tumor fragments (~30 mm^3^ size) were implanted subcutaneously at the flank area of female BALB/c nude mice (BK Laboratory Animal Co., Ltd.; Shanghai, China).

Animal studies using HBCx8 PDX model were conducted at XenTech (Cerfe, Evry, France). The animal care and housing were in accordance with French regulatory legislation concerning the protection of laboratory animals. To establish the model, tumor fragments (~20 mm^3^ size) were implanted subcutaneously in the interscapular region of female athymic nude mice (Envigo; Gannat, France).

When tumors reached the desired volume (~200 mm^3^), animals were divided into groups of n = 5–10 with a similar distribution of tumor volumes and received intravenous dose(s) of vehicle (20 mM histidine acetate, 240 mM sucrose, 0.02% polysorbate-20, pH 5.5) or ADCs through the tail vein. The treatment information was not blinded during measurement. Tumors were measured in two dimensions (length and width) using calipers and tumor volume was calculated using the formula: Tumor size (mm^3^) = 0.5 x (length x width x width). Changes in body weights were reported as a percentage relative to the starting weight. Tumor sizes and mouse body weights were recorded twice weekly over the course of the study. Mice whose tumor volume exceeded 2000 mm^3^ or whose body weight loss was 20% of their starting weight were promptly euthanized per IACUC guidelines.

Data were analyzed using R statistical software system (R Foundation for Statistical Computing; Vienna, Austria), and a mixed modeling was fit within R using the nlme package.^57^ Cubic regression splines were used to fit a non-linear profile to the time courses of body weight change or log2 tumor volume at each dose level. These non-linear profiles were then related to dose within the mixed model. This approach addresses both repeated measurements and modest dropouts due to any non-treatment-related removal of animals before study end. Results were plotted in natural scale as fitted body weight change or tumor volume of each group over time.

### Cell viability assays

Cells were seeded in 384-well plates, grown for 24 h, and treated with either unconjugated compound or ADC. After 3 d (small molecules) or 5 d (ADCs) of continuous drug incubation, the cell viability was determined using Promega CellTiter-Glo luminescent reagent (G7570), which measures the adenosine triphosphate level (an indirect measure of cell number). The luminescent intensity was measured using a PerkinElmer Envision reader. The relative cell viability was calculated by normalizing to non-drug treatment control and was graphed using KleidaGraph software package. IC50 values were determined to show concentration to obtain 50% of the maximum cell killing.

### Radiochemistry

Iodine-125 (^125^I) was obtained as sodium iodide in 0.1 N sodium hydroxide from Perkin Elmer. Indirect iodinations were done as previously described using 1 mCi of ^125^I (3 µL) to randomly iodinate tyrosine residues at a specific activity of ~5-8 mCi/mg with ^125^I using iodogen tubes (Pierce). Indium-111 (^111^In) was obtained as indium chloride in 0.05 N hydrogen chloride from BWX Technologies, Inc. Radiosynthesis of ^111^In-labeled antibodies (5–9 mCi/mg) was achieved through incubation of ^111^InCl_3_ and 1,4,7,10-tetraazacyclododecane-1,4,7,10-tetraacetic acid (DOTA)-conjugated (site directed through cysteines) monoclonal antibody in 0.1 mol/L HEPES pH 5.5 at 37°C. Purification of all radioimmunoconjugates was achieved using NAP5 columns equilibrated in PBS and confirmed by radio-size-exclusion chromatography.

### Tissue distribution

All animal experiments were conducted in accordance with an IACUC. Biodistribution studies were performed in female SCID-BG mice bearing HCC1569x2 xenografts. Tumors inoculated into the mammary fat pads were allowed to reach 250–300 mm^3^ prior to random assignment into groups (n = 8). To prevent thyroid sequestration of ^125^I, 100 µL of 30 g/L of sodium iodide was intraperitoneally administered 1 h and 24 h prior to dosing. Each mouse received a single intravenous bolus consisting of ^125^I- and ^111^In-labeled antibodies (5 µCi of each isotope); with additional N-ethyl-maleimide-capped parent ThioMab for a total dose of 1 mg/kg. Blood samples were collected from each animal at 1, 24, and 72 h to derive plasma and whole blood antibody concentrations. At 24 and 72 h, tissue samples (n = 4) were promptly collected by terminal organ harvest, rinsed with PBS and counted for radioactivity using a 1480 WIZARD Gamma Counter in the energy windows for ^111^In (245 keV; decay t_1/2_ 2.8 d) and ^125^I (35 keV; decay t_1/2_ 59.4 d) with automatic background and decay correction. Data were graphed and analyzed using Prism (Graphpad).

### Pharmacokinetics

Female SCID.bg mice received single intravenous doses (0.1, 1 or 10 mg/kg) of anti-Ly6E or anti-Ly6Ev2 dissolved in a histidine acetate 20 mM, sucrose 240 mM, tween-20 0.02%, pH 5.5 formulation buffer. Blood samples were obtained retro-orbitally (left, right eyes) or by cardiac puncture at the terminal time point (n = 3 at each time point; ~125 uL collection volume) and processed for serum at 10 min, 1 h, 6 h, 1 d, 2 d, 3 d, 7 d, 14 d, and 21 d in a rotated schedule. Pharmacokinetic parameters were estimated by analyzing plasma concentration vs. time data using noncompartmental approach (WinNonlin, Pharsight Corp., Mountain View, CA). Plasma concentration vs. time data were naïve pooled together (sparse sampling approach) to provide pharmacokinetic parameter estimations. Parameters calculated included the maximum concentration (Cmax); area under the plasma concentration–time curve from time = 0 to infinity (AUC_0-∞_); clearance (CL) and volume of distribution (Vz).

## Supplementary Material

Supplemental Material

## List of abbreviations

Antibody-drug conjugates (ADCs), cyclopropyl benzoindol-4-one (CBI), Chinese hamster ovary (CHO), drug-to-antibody ratio (DAR), light chain (LC), lymphocyte antigen 6E (Ly6E), monomethyl auristatin E (MMAE), pyrrolobenzodiazepine (PBD), pyrrolobenzodiazepine dimer mono-amide (PBD-MA), phosphate-buffered saline (PBS), *P*-glycoprotein (Pgp), virus-like particles (VLPs), tumor-mediated drug disposition (TMDD)
