# Supplementary material for: Preclinical optimization of Ly6E-targeted ADCs for increased durability and efficacy of anti-tumor response
Source: MAbs. 2020 Dec 31;13(1):1862452. doi: 10.1080/19420862.2020.1862452 (PMC7784788; doi:10.1080/19420862.2020.1862452)
Supplement: Supplemental Material [file KMAB_A_1862452_SM5877.docx]

**­­Supplementary material**

**Supplementary Table 1**

*Pharmacokinetic parameters for anti-Ly6E antibodies*. Values composite from naïve pooling of animals. Reported parameter variability in Cmax represents standard error (SE) and is a result of naïve pool approach with non-compartmental analysis (NCA).

**­­**A.

B.


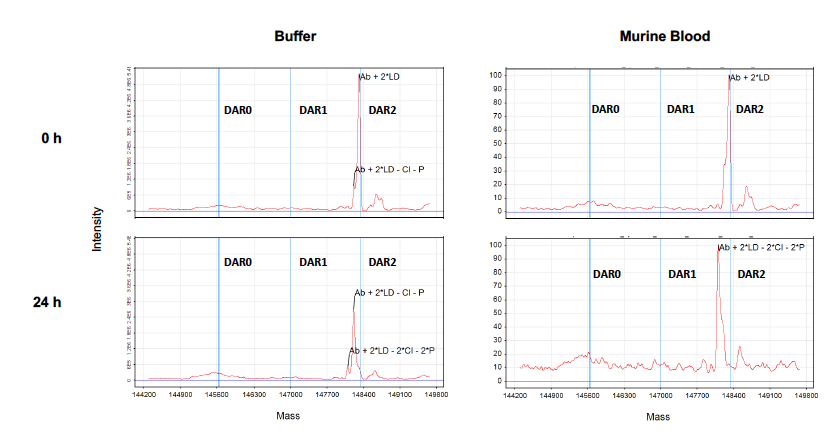


**Supplementary Figure 1:** *seco-CBI conjugation and stability:* *(A)* Structure of CBI-linker drug. *(B)* ADC was added to buffer or mouse whole blood and incubated at 37 °C for 24 h. Samples were analyzed by affinity capture LC-MS to assess the extent of drug deconjugation from the antibody.

**Supplementary Figure 2: ­** *In vivo efficacy of anti-Ly6E-CBI in tumor xenograft models of breast cancer* (Left) BR-05-014E, *and (*Middle*)* HBCx8, *and lung cancer*: (Right) NCI-H1781. Numbers next to traces indicate dose of each conjugate (in mg/kg) that was administered once IV at day 0. Cubic spline fitted tumor volumes are plotted for each treatment group (n=5-7/group).

**
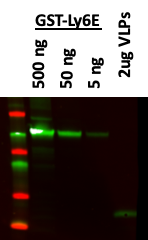
**

**Supplementary Figure 3:** *Quantification of Ly6E in VLPs*. Recombinant GST-Ly6E (Novus H00004061-P01-10ug) (5-500 ng) and extracellular vesicles derived from cells overexpressing Ly6E containing 2 μg of total protein were separated by SDS-PAGE, transferred to nitrocellulose and analyzed by immunoblot, probing with anti-Ly6E (Sigma SAB1401233). Fluorescent quantitation of the Ly6E bands estimates 10-15 ng of Ly6E per 2 μg of VLP protein.

A.

B.


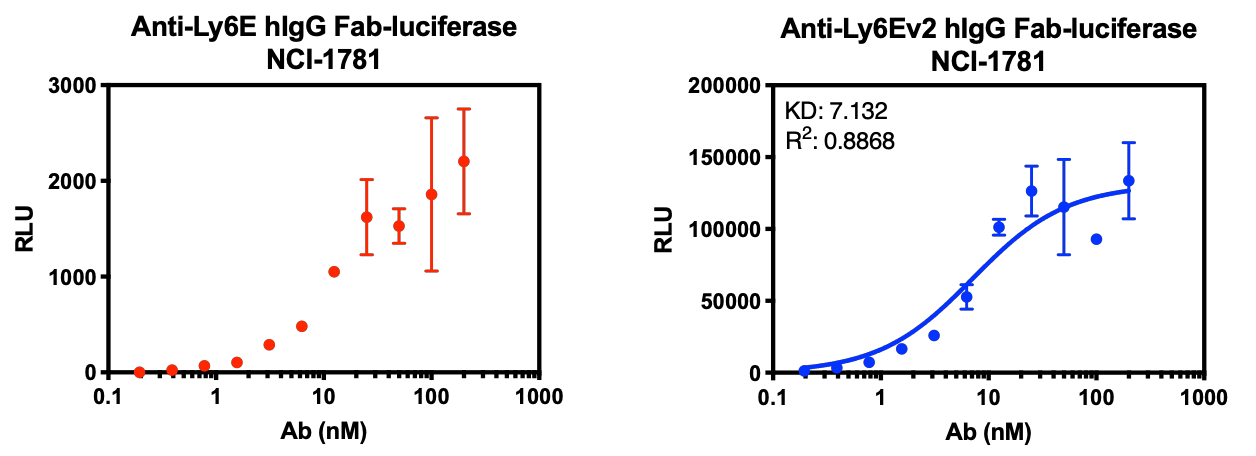


C.


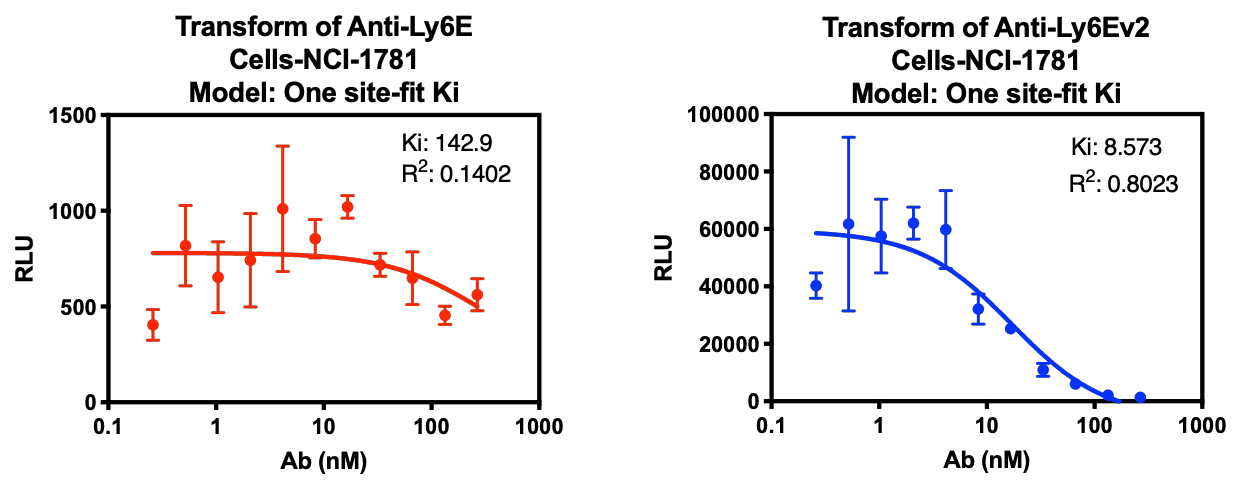


**Supplementary Figure 4**: *Characterization of binding of humanized anti-Ly6Ev2.* *(A)* Anti-Ly6Ev2 was humanized via CDR graft into the closest human germline sequence and introduction of rat Vernier residues. Chimeric and humanized versions of anti-Ly6Ev2 were incubated with PC3 cells overexpressing Ly6E, washed and analyzed for bound antibody by flow cytometry. *(B)* Anti-Ly6E-Fab-luciferase fusions were incubated with NCI-H1781 cells at 4 °C for four hours, washed, and bound antibody was determined by addition of luciferase substrate. Fits were determined using non-linear fit models in Prism. *(C)* Mixtures of Anti-Ly6E-Fab-luciferase fusions (20 nM) and Anti-Ly6E-Fabs (0-200 nM) were incubated with NCI-H1781 cells at 4 °C for four hours, washed, and bound antibody was determined by addition of luciferase substrate. Fits were determined using one site Ki models in Prism.

**Supplementary Figure 5:** *Characterization of anti-Ly6E binding*. a. Cell lysates from multiple different cell lines were analyzed by immunoblot, staining with polyclonal rabbit anti-Ly6E (GEN-93-8-1). *b.* Flow cytometry analysis of cell lines characterized as Ly6E positive by immunoblot. Points represent the average of 2 replicate wells. *c.* Flow cytometry analysis of cell lines characterized as Ly6E negative by immunoblot. Points represent the average of 2 replicate wells.

**Supplementary Figure 6:** *Fluorescent microscopy characterization of anti-Ly6E antibodies*. *(A)* Kuramochi cells were continuously exposed to 2 ug/ml of antibody as described in **Figure 4a** and total cell surface antibody was quantified. *(B)* Kuramochi cells were pulsed with 2 ug/ml of antibodies as described in **Figure 4c.** Lysosome colocalized antibody was quantified.

**
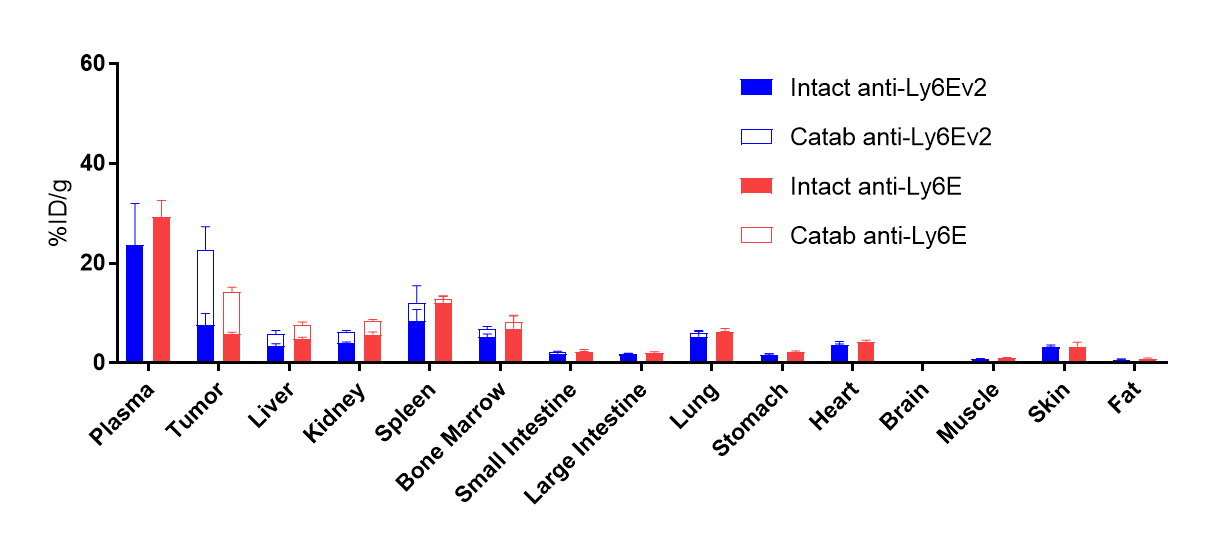
Supplementary Figure 7:** *Biodistribution of anti-Ly6E antibodies at 24h.* HCC1569x2 tumor bearing mice received a single intravenous bolus of radiolabeled Ly6E targeting antibodies (1 mg/kg), anti-Ly6E (red) or anti-Ly6Ev2 (blue) Tissue distribution is shown at 24 hours, with values are reported as a percentage of injected radioactive dose normalized to gram of dry blotted tissue (%ID/g) to convey changes in enrichment. Filled bars represent intact antibody values, while hollow bars represent catabolized values. The total value of the stacked bars represents intact and catabolized exposure up to that time point. The graph shows the mean and SD for each group with n=4.

**Supplementary Figure 8*.*** *Tolerability of anti-Ly6Ev2-SN36325 and anti-Ly6Ev2-PBD-monoamide in the efficacy studies shown in* ***Figure 6***. (A, C) HCC1569X2 breast cancer, and (B, D) SW900 lung cancer. Numbers next to traces indicate dose of each conjugate (in mg/kg) that was administered once IV at day 0. Cubic spline fitted body weight changes are plotted for each treatment group (n=5-8/group).

**Supplementary Figure 9:** *Structure of PBD-MA*. Chemical structures of the PBD-monoamide (*top*) and the PBD-monoamide linker drug (*bottom*). The linker drug can be attached to antibodies containing free thiols via disulfide exchange.

**Supplementary Methods**

**Synthesis of PBD-monoamide linker drug**

**Scheme**

**Experimental**

***General procedure for preparation of compound 4:***

To a solution of compound **3** (2.0 g, 2.92 mmol) in anhydrous DCM (40 mL) was added imidazole (1.19 g, 17.53 mmol), followed by TBSCl (881 mg, 5.84 mmol). The reaction mixture was stirred at 40 ^o^C for 3 h. The mixture was diluted with DCM (50 mL), washed with H_2_O (30 mL × 3), dried over Na_2_SO_4_, filtered, and concentrated. The residue was purified by chromatography on silica (solvent gradient: 0 - 3.3 % MeOH in DCM) to afford compound **4** (1.2 g, 50.9%) as a yellow solid.

LCMS (5-95, AB, 1.5 min): R_T_ = 1.064 min, m/z = 799.3[M+H]^+^.

***General procedure for preparation of compound 5:***

To a solution of compound **4** (800 mg, 1.00 mmol) in anhydrous DCM (100 mL) was added DMP (1.70 g, 4.01 mmol). After the reaction mixture was stirred at r.t. for 2 h, it was filtered, diluted with EtOAc (400 mL), washed with sat. Na_2_SO_3_ solution (100 mL), sat. Na_2_SO_3_/NaHCO_3_ solution (1:1, 100 mL) and brine (100 mL). The organic layer was dried over Na_2_SO_4_, filtered, and concentrated to afford crude aldehyde. The crude aldehyde was dissolved in t-BuOH (40 mL)/H_2_O (20 mL). 2-Methyl-2-butene (30 mL) and NaH_2_PO_4_ (480 mg, 4.00 mmol) were added successively at r.t. After it was stirred at r.t. for 0.5 h, NaClO_2_ (543 mg, 6.00 mmol) was added and the mixture was stirred at r.t. for additional 1 h. The mixture was diluted with EtOAc (200 mL), washed with water (50 mL) and brine (50 mL). The organic layer was dried over Na_2_SO_4_, filtered, and concentrated in vacuo to afford compound **5** (700 mg, 80.8 %) as crude product. LCMS (5-95, AB, 1.5 min): R_T_ = 1.062 min, m/z = 813.3[M+H]^+^.

***General procedure for preparation of compound 6:***

To a solution of compound **5** (700 mg, 0.86 mmol) in DMF (10 mL) was added K_2_CO_3_ (595 mg, 4.31 mmol), followed by MeI (1.2 g, 8.60 mmol). After the reaction mixture was stirred at r.t. overnight, it was filtered, the filtrate was diluted with EtOAc (200 mL), washed with brine (40 mL × 5), dried over Na_2_SO_4_, filtered, and concentrated in vacuo to afford compound **6** (700 mg, 98.3 %) as crude product. LCMS (5-95, AB, 1.5 min): R_T_ = 1.108 min, m/z = 827.4[M+H]^+^.

***General procedure for preparation of compound 7:***

To a solution of compound **6** (700 mg, 0.85 mmol) in EtOH/H_2_O (20 mL/10 mL) was added iron powder (474 mg, 8.50 mmol), followed by NH_4_Cl (910 g, 17.0 mmol). The reaction mixture was stirred at 70 ^o^C for 2 h. The mixture was filtered, and the filtrate was concentrated in vacuo to remove EtOH, and the water slurry was extracted with EtOAc (50 mL × 3). The combined EtOAc layers were dried over Na_2_SO_4_, filtered, and concentrated in vacuo. The residue was purified by chromatography on silica (solvent gradient: 0 - 3.3 % MeOH in DCM) to afford compound **7** (600 mg, 96.6 %) as a yellow solid. LCMS (5-95, AB, 1.5 min): R_T_ = 0.971 min, m/z = 735.3[M+H]^+^.

^1^H NMR (400 MHz, CDCl_3_) *δ* 8.04 (br s, 1H), 7.41 (s, 1H), 6.75 (s, 1H), 6.43 (s, 1H), 6.24 (s, 1H), 5.17 - 5.11 (m, 2H), 4.97 - 4.90 (m, 2H), 4.44 - 4.34 (m, 2H), 4.23 - 4.17 (m, 3H), 3.99 (t, *J* = 6.8 Hz, 5H), 3.90 (s, 3H), 3.76 (s, 4H), 3.62 (br.s, 1H), 3.47 - 3.45 (d, *J* = 16.0 Hz, 1H), 2.85 - 2.81 (m, 1H), 2.74 - 2.64 (m, 2H), 1.94 - 1.86 (m, 4H), 1.66 - 1.62 (m, 2H), 0.87 (s, 9H), 0.02 (s, 6H).

***General procedure for preparation of compound 9:***

After triphosgene (121 mg, 0.41 mmol) in anhydrous DCM (15 mL) was cooled at 0 ^o^C, compound **8** (201 mg, 0.82 mmol) and pyridine (258 mg, 3.27 mmol) in anhydrous DCM (15 mL) was added dropwise. The reaction solution was stirred at 0^o^C for 10 min. After the solution was concentrated in vacuo, and the residue was dissolved in anhydrous DCM (15 mL), and added into a solution of compound **7** (300 mg, 0.41 mmol) and DIEA (206 mg, 2.04 mmol) in anhydrous DCM (15 mL) at 0 ^o^C. The reaction mixture was stirred at 0 ^o^C for 1 h, and diluted with DCM (100 mL), washed with H_2_O (40 mL × 5), dried over Na_2_SO_4_, filtered, and concentrated in vacuo. The residue was purified chromatography on silica to afford compound **9** (300 mg, yield: 72.7 %) as crude product. LCMS (5-95, AB, 1.5 min): R_T_ = 1.133 min, m/z = 1007.3[M+H]^+^.

***General procedure for preparation of compound 8:***

To a solution of compound **9** (300 mg, 0.30 mmol) in THF/H_2_O (6 mL/3 mL) was added HOAc (9 mL), and the mixture was stirred at 40 ^o^C overnight. Then the solution was concentrated in vacuo to remove the solvent, and the residue was diluted with EtOAc (100 mL), washed with H_2_O (30 mL × 5), dried over Na_2_SO_4_, filtered, and concentrated. The residue was purified by pre-TLC (DCM/MeOH = 12:1) to afford compound **10** (250 mg, yield: 91.2 %) as a yellow solid. LCMS (5-95, AB, 1.5 min): R_T_ = 0.899 min, m/z = 893.2[M+H]^+^.

***General procedure for preparation of GNT_B343_541:***

To a solution of compound **10** (200 mg, 0.22 mmol) in anhydrous DCM (20 mL) was added DMP (114 mg, 0.27 mmol). The reaction mixture was stirred at r.t. for 1 h. Then the mixture was diluted with DCM (50 mL), filtered. The filtrate was washed with Na_2_SO_3_ (30 mL × 3), dried over Na_2_SO_4_, filtered and concentrated in vacuo. The residue was purified by prep-TLC (DCM/MeOH = 20:1) to afford the crude product. The crude product was further purified by prep-HPLC (FA) to afford **PBD-monoamide linker drug** (65 mg, 33.2%) as pale yellow solid. LCMS (5-95, AB, 1.5 min): R_T_ = 0.871 min, m/z = 891.2[M+H]^+^.

^1^H NMR (400 MHz, CDCl*_3_*) *δ* 9.21 (s, 1H), 8.72 (s, 1H), 8.26 (d, *J* = 8.0 Hz, 1H), 7.56 (d, *J* = 8.4 Hz, 1H), 7.39 (s, 1H), 7.24 (s, 1H), 6.83 (s, 1H), 6.47 (s, 1H), 5.58 (d, *J* = 9.2 Hz, 1H), 5.14 - 5.09 (m, 4H), 4.91 (br, 1H), 4.44 – 4.39 (m, 1H), 4.30 - 4.19 (m, 4H), 4.14 - 4.05 (m, 2H), 3.97 - 3.86 (m, 10H), 3.63 (t, *J* = 8.8 Hz, 1H), 3.46 – 3.42 (m, 1H), 3.14 – 3.12 (m, 1H), 2.94 - 2.79 (m, 2H), 2.72 – 2.68 (m, 1H), 1.86 (br s, 4H), 1.57 (br s, 2H), 1.13 (d, *J* = 6.4 Hz, 3H).
